# Supplementary material for: Interphase chromosome conformation is specified by distinct folding programmes inherited through mitotic chromosomes or the cytoplasm
Source: Nat Cell Biol. 2025 Dec 22;28(1):82–97. doi: 10.1038/s41556-025-01828-1 (PMC12807859; doi:10.1038/s41556-025-01828-1)

nature cell biology

Supplementary information

https://doi.org/10.1038/s41556-025-01828-1

Interphase chromosome conformation is specified by distinct folding programmes inherited through mitotic chromosomes or the cytoplasm

In the format provided by the authors and unedited

Supplementary Information

SUPPLEMENTARY FIGURES LIST

Supplementary Figure 1: FACS strategy
Supplementary Figure 2: Hi-C samples and reproducibility
Supplementary Figure 3: Enrichment based detection of microcompartment domains (MCDs)

SUPPLEMENTARY FIGURE LEGENDS

Supplementary Figure 1: FACS strategy

a. Flow cytometry filtering procedure used to isolate prometaphase arrested cells fixed for Hi-C and stained with Propidium Iodide (PI). Live cells were selected based on forward and side scatter (FSC, SSC). An example of DNA content (PI) Area vs Width for selected live cells is shown. Sorting gates were set, as for the example (right histogram), and re-adjusted throughout sorting to avoid G2 contamination.
b. FACS filtering procedure used to isolate G1 cells 5h after prometaphase release fixed for Hi-C and stained with Propidium Iodide (PI). Live cells were selected based on FSC and SSC Area. An example of DNA content (PI) Area vs Width for selected live cells is shown. Sorting gates were set, as for the example (right histogram), and re-adjusted throughout sorting to avoid G2 contamination.

Supplementary Figure 2: Hi-C samples and reproducibility

a. Sequencing summary statistics for generated Hi-C samples. Samples are grouped as following: RanGAP1 control and depletion samples across timepoints, Nup93 control and depletion samples in G1 (5h and 10h), samples related to the RanGAP1 G1-depletion experiment, and samples related to BET protein inhibition. The total number of sequenced PE reads, percentage of uniquely mapped paired end reads, cis-chromosomal vs. trans-chromosomal percentage breakdown of uniquely mapped pairs, and pooled naming used for Hi-C related analyses, are depicted (from left to right).
b. Comparison of EV1 values from Eigenvector decompositions of 25kb binned Hi-C data from control or Auxin-treated RanGAP1-AID or Nup93-AID cells released to early G1.
c. PCA analysis of the leading eigenvectors (EV1 at 25 kb resolution) across all Hi-C samples. Samples used to define the principal components are depicted in red, the remaining samples were projected onto the derived components (depicted in black) including individual replicates (depicted in grey and connected to their corresponding pooled sample via black lines). PC1 (explaining >50% of variance) clearly related to the cell cycle stage, whereas PC2 (explaining >20%) clearly separates depletion conditions from the controls (5hr G1) in both pooled and individual replicate samples.
d. PCA analysis of the insulation tracks (at 10 kb resolution, 100 kb diamond size) across all Hi-C samples and coloured as for (c). PC1 (explaining 50% of variance) clearly separates depletion conditions from the controls (5hr G1), whereas PC2 (explaining 25%) relates to the cell cycle stage. Individual replicates of the samples demonstrate concordance according to this metric in pooled and individual replicate samples.

Supplementary Figure 3: Enrichment based detection of microcompartment domains (MCDs)

a. An example of a “flattened” Hi-C contact map (observed/expected) from the RanGAP1-AID-depleted G1 sample. MCD-MCD interactions are enriched above the expected level and are clearly visible.
b. First step of the MCD detection procedure: Convolution of the contact map with the selected kernels (M, V, H) and thresholding enriched pixels, such that M > 2*(V or H). Enriched pixels are depicted in black, zoom-in is provided on the right.
c. Second step of the MCD detection procedure: Spatial clustering of enriched pixels in order to filter out singleton and other spurious calls. Large clusters that were kept are highlighted with yellow bounding boxes on the selected region of the contact map and the zoom-in (right).
d. Third step of the MCD detection procedure: Enriched pixels from the kept clusters are used to calculate “coverage” (i.e., the number of enriched pixels that overlap a given genomic bin). “Strong” anchors are apparent as prominent peaks on the coverage track for the selected genomic region (center).
e. Fourth step of the MCD detection procedure: 1D peak detection applied to the coverage track results in the detected MCDs shown for the selected genomic region in the center panel. Borders of the peak footprints are depicted in gray, while summits are shown in yellow.
f. Resulting MCDs are used to construct an all-by-all grid or “microcompartment”, as shown in the center panel. Each instance of MCD-MCD interaction is depicted using a black bounding box. A typical way to explore a local pattern of interaction around MCDs is to pile them as shown on the right and to calculate the average signal.

Supplementary Figure 1: FACS strategy

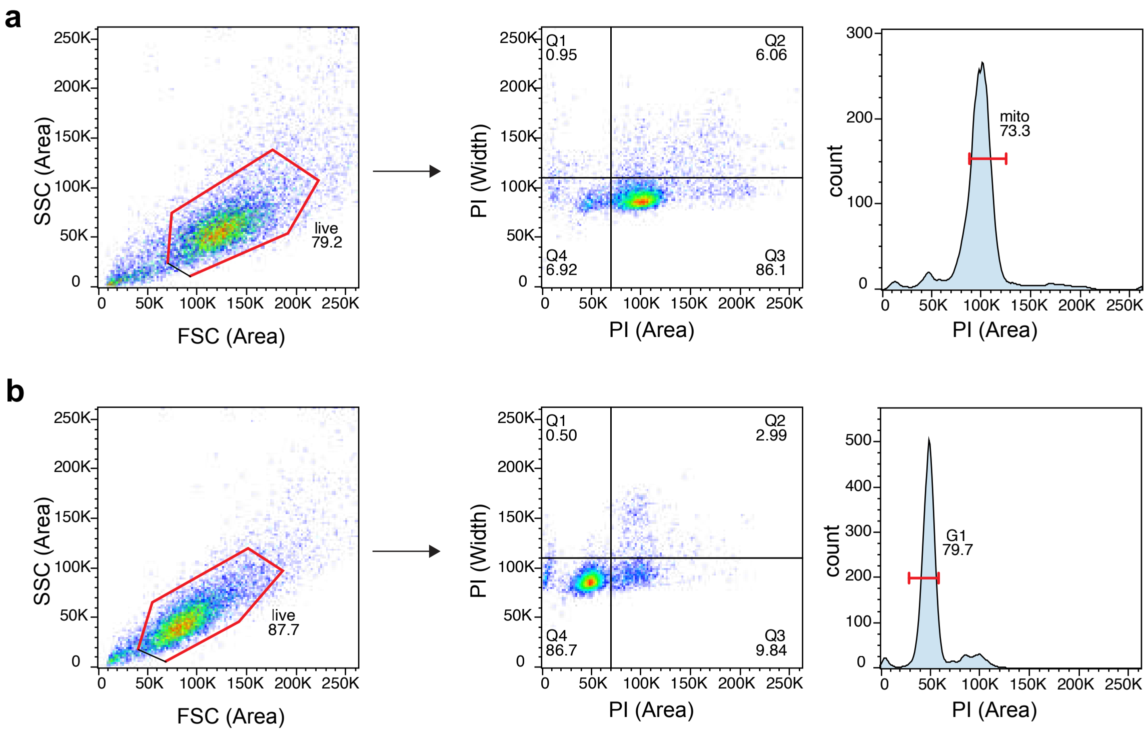

Supplementary Figure 2: Hi-C samples and reproducibility

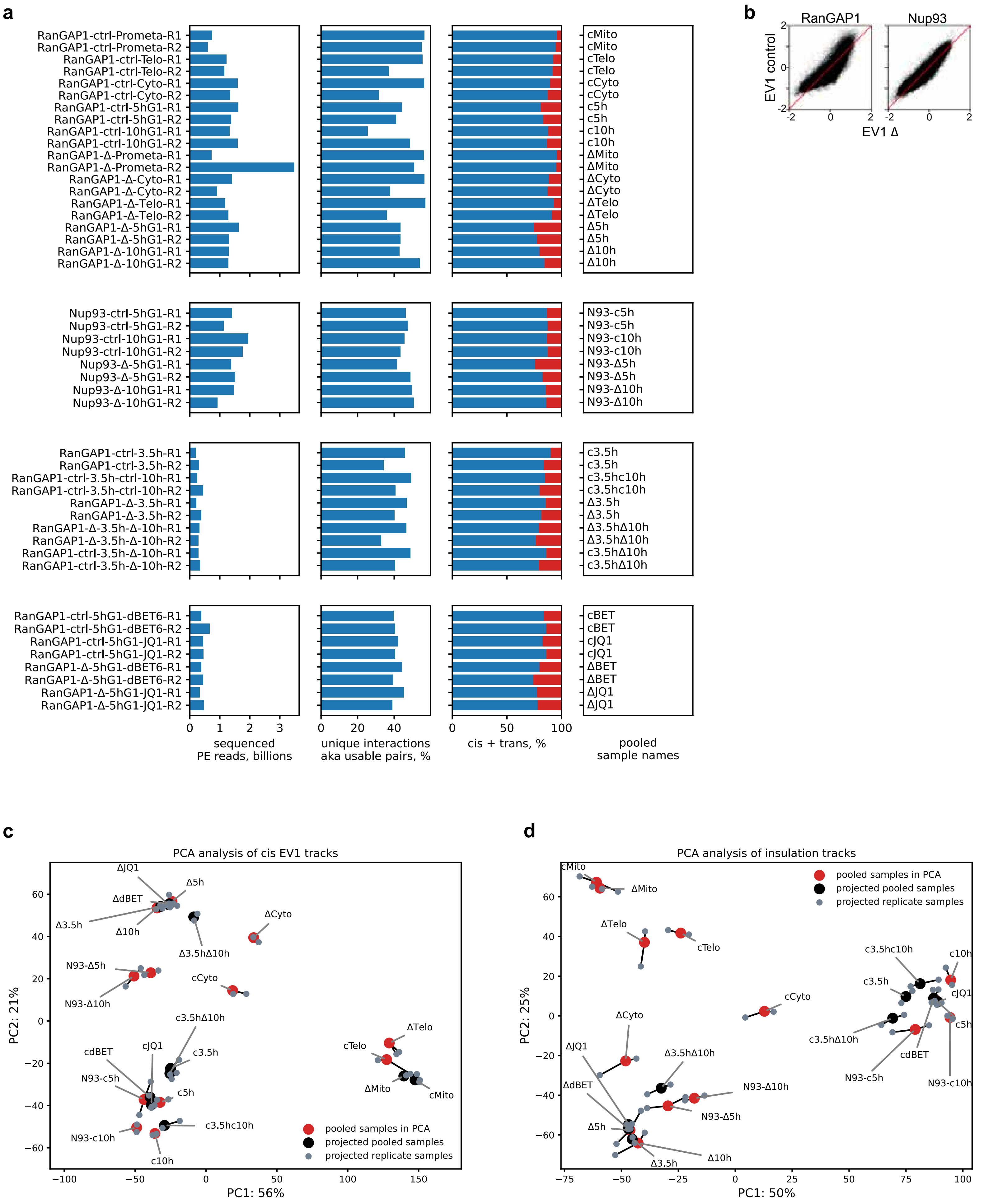

Supplementary Figure 3: Enrichment based detection of microcompartment domains (MCDs)

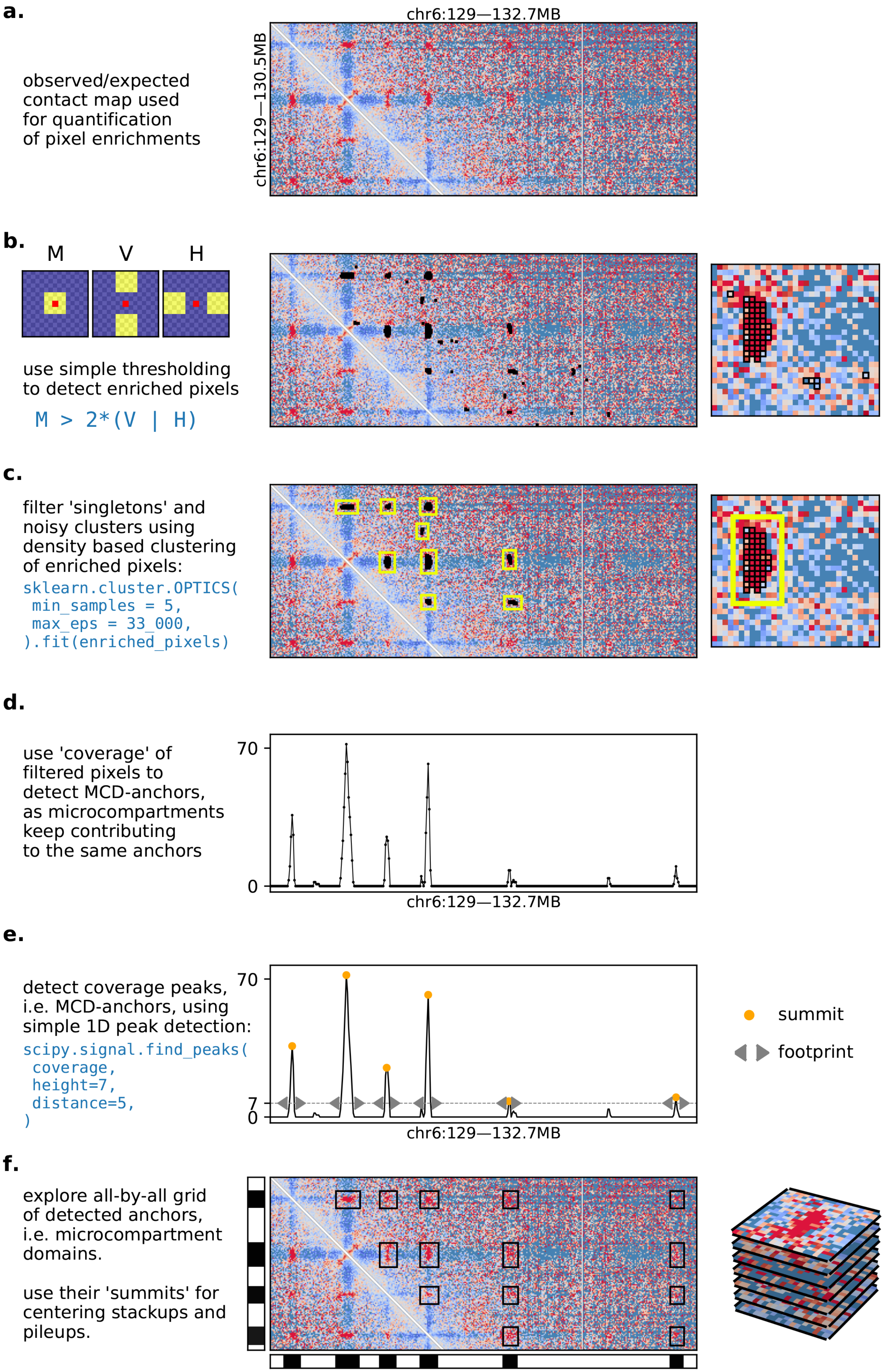

Supplement: Supplementary file 1 — Supplementary Figs.1–3. [file 41556_2025_1828_MOESM1_ESM.pdf]
