# Supplementary material for: Interphase chromosome conformation is specified by distinct folding programmes inherited through mitotic chromosomes or the cytoplasm
Source: Nat Cell Biol. 2025 Dec 22;28(1):82–97. doi: 10.1038/s41556-025-01828-1 (PMC12807859; doi:10.1038/s41556-025-01828-1)
Supplement: Supplementary file 2 — Reporting Summary [file 41556_2025_1828_MOESM2_ESM.pdf]

nature portfolio

Corresponding author(s): Job Dekker

Last updated by author(s): 10/24/2025

Reporting Summary

Nature Portfolio wishes to improve the reproducibility of the work that we publish. This form provides structure for consistency and transparency in reporting. For further information on Nature Portfolio policies, see our Editorial Policies and the Editorial Policy Checklist.

Statistics

For all statistical analyses, confirm that the following items are present in the figure legend, table legend, main text, or Methods section.

n/a	Confirmed	
☐	☒	The exact sample size (*n*) for each experimental group/condition, given as a discrete number and unit of measurement
☐	☒	A statement on whether measurements were taken from distinct samples or whether the same sample was measured repeatedly
☐	☒	The statistical test(s) used AND whether they are one- or two-sided<br>*Only common tests should be described solely by name; describe more complex techniques in the Methods section.*
☒	☐	A description of all covariates tested
☒	☐	A description of any assumptions or corrections, such as tests of normality and adjustment for multiple comparisons
☐	☒	A full description of the statistical parameters including central tendency (e.g. means) or other basic estimates (e.g. regression coefficient) AND variation (e.g. standard deviation) or associated estimates of uncertainty (e.g. confidence intervals)
☐	☒	For null hypothesis testing, the test statistic (e.g. *F*, *t*, *r*) with confidence intervals, effect sizes, degrees of freedom and *P* value noted<br>*Give P values as exact values whenever suitable.*
☒	☐	For Bayesian analysis, information on the choice of priors and Markov chain Monte Carlo settings
☒	☐	For hierarchical and complex designs, identification of the appropriate level for tests and full reporting of outcomes
☒	☐	Estimates of effect sizes (e.g. Cohen's *d*, Pearson's *r*), indicating how they were calculated

Our web collection on statistics for biologists contains articles on many of the points above.

Software and code

Policy information about availability of computer code

Data collection

NovaSeq Control Software 1.8.0 (Hi-C) and Illumina BaseSpace/DRAGEN secondary analysis v4.0 (ATACseq, Cut&Run, SLAMseq)

Data analysis

Code used in this study can be found on Github:
https://github.com/dekkerlab/inherited-folding-programs

We also used the following scripts/software:

https://github.com/open2c/distiller-nf v0.3.4
https://github.com/open2c/pairtools v1.0.2
https://github.com/open2c/cooler v0.8.11
https://github.com/open2c/cooltools v0.7.0
https://github.com/open2c/bioframe v0.3.1
https://github.com/open2c/coolpuppy v0.9.5
pybbi v0.3.2
clodius v0.3.5
bedtools v2.29.2
matplotlib v3.5.2
scaffold v5.3.3
MaxQuant v2.6.5.0
nf-core/ataqseq v2.1.0
nf-core/RNAseq v3.15.0

https://github.com/loosolab/TOBIAS
macs3 v3.0.2
scikit-learn v0.23.0
scipy v1.5.2
R v4.1.0
DESeq2 v3.15
Image Lab 6.0.1 builder 34
FlowJo v10
HISAT-3N

https://github.com/open2c/open2c_vignettes/blob/main/sparse_eigendecomp.ipynb
https://github.com/nf-core/atacseq

For manuscripts utilizing custom algorithms or software that are central to the research but not yet described in published literature, software must be made available to editors and reviewers. We strongly encourage code deposition in a community repository (e.g. GitHub). See the Nature Portfolio guidelines for submitting code & software for further information.

Data

Policy information about availability of data

All manuscripts must include a data availability statement. This statement should provide the following information, where applicable:

- Accession codes, unique identifiers, or web links for publicly available datasets
- A description of any restrictions on data availability
- For clinical datasets or third party data, please ensure that the statement adheres to our policy

The datasets generated in this publication have been deposited in NCBI's Gene Expression Omnibus as a SuperSeries accessible through GEO accession number GSE278023, consisting of GSE277875 (Hi-C), GSE277731 (ATAC-seq), GSE308844 (Cut&Run), and GSE309609 (SLAMseq). The following published datasets were used in this study (Supplementary Table 3): GSE132363, GSE178593, and GSE214012. The mass spectrometry proteomics data have been deposited to the ProteomeXchange Consortium via the PRIDE (Perez-Riverol et al. 2022) partner repository with the dataset identifier PXD056346. Source data are provided with this paper. All other data supporting the findings of this study are available from the corresponding author on reasonable request.

GEO accession GSE278023: reviewer token is qtcpqeowhrovfon.
ProteomeXchange #PXD056346: Username: reviewer_pxd056346@ebi.ac.uk Password: aGGcVWQJR8gb

Research involving human participants, their data, or biological material

Policy information about studies with human participants or human data. See also policy information about sex, gender (identity/presentation), and sexual orientation and race, ethnicity and racism.

Reporting on sex and gender	*Use the terms sex (biological attribute) and gender (shaped by social and cultural circumstances) carefully in order to avoid confusing both terms. Indicate if findings apply to only one sex or gender; describe whether sex and gender were considered in study design; whether sex and/or gender was determined based on self-reporting or assigned and methods used. Provide in the source data disaggregated sex and gender data, where this information has been collected, and if consent has been obtained for sharing of individual-level data; provide overall numbers in this Reporting Summary. Please state if this information has not been collected. Report sex- and gender-based analyses where performed, justify reasons for lack of sex- and gender-based analysis.*
Reporting on race, ethnicity, or other socially relevant groupings	*Please specify the socially constructed or socially relevant categorization variable(s) used in your manuscript and explain why they were used. Please note that such variables should not be used as proxies for other socially constructed/relevant variables (for example, race or ethnicity should not be used as a proxy for socioeconomic status). Provide clear definitions of the relevant terms used, how they were provided (by the participants/respondents, the researchers, or third parties), and the method(s) used to classify people into the different categories (e.g. self-report, census or administrative data, social media data, etc.) Please provide details about how you controlled for confounding variables in your analyses.*
Population characteristics	*Describe the covariate-relevant population characteristics of the human research participants (e.g. age, genotypic information, past and current diagnosis and treatment categories). If you filled out the behavioural & social sciences study design questions and have nothing to add here, write "See above."*
Recruitment	*Describe how participants were recruited. Outline any potential self-selection bias or other biases that may be present and how these are likely to impact results.*
Ethics oversight	*Identify the organization(s) that approved the study protocol.*

Note that full information on the approval of the study protocol must also be provided in the manuscript.

Field-specific reporting

Please select the one below that is the best fit for your research. If you are not sure, read the appropriate sections before making your selection.

☒ Life sciences ☐ Behavioural & social sciences ☐ Ecological, evolutionary & environmental sciences

For a reference copy of the document with all sections, see nature.com/documents/nr-reporting-summary-flat.pdf

Life sciences study design

All studies must disclose on these points even when the disclosure is negative.

Sample size	No statistical methods were used to predetermine sample size. The use of two replicates is common practice in molecular biology and genomics, given the cost and statistics are needed to be well balanced (PMID:25317452).
Data exclusions	No data were excluded from these analyses since all the data generated in this study passed quality control.
Replication	All experiments have at least two independent biological replicates. All findings described in the manuscript were confirmed in all individual replicates.
Randomization	Randomization of this study was not necessary as we did not allocate datasets into experimental groups.
Blinding	All analyses did not require blinding because results were directly linked with the data and this is neither a clinical study with large cohorts nor a genetics study with large numbers of samples.

Reporting for specific materials, systems and methods

We require information from authors about some types of materials, experimental systems and methods used in many studies. Here, indicate whether each material, system or method listed is relevant to your study. If you are not sure if a list item applies to your research, read the appropriate section before selecting a response.

Materials & experimental systems

n/a	Involved in the study	
☐	☒	Antibodies
☐	☒	Eukaryotic cell lines
☒	☐	Palaeontology and archaeology
☒	☐	Animals and other organisms
☒	☐	Clinical data
☒	☐	Dual use research of concern
☒	☐	Plants

Methods

n/a	Involved in the study	
☒	☐	ChIP-seq
☐	☒	Flow cytometry
☒	☐	MRI-based neuroimaging

Antibodies

Antibodies used

commercial antibodies used in this study are listed in the relevant supplementary methods.

Western blotting:
Primary antibodies: 1:500 mouse anti-RanGAP1 (OTI1B4, Novus Biologicals, NBP2-02623), 1:500 mouse anti-Nup93 (F-2, Santa Cruz Biotechnology, sc-374400), 1:1000 rabbit anti-vinculin (EP18185, Abcam, ab129002), 1:1000 rabbit anti-BRD4 (E2A7X, Cell Signaling 13440), 1:1000 rabbit anti-BRD2 (EPR7642, ab139690). Secondary antibodies: 1:1000 goat anti-mouse IgG-HRP (Cell Signaling 7076), 1:1000 goat anti-rabbit IgG-HRP (Cell Signaling 7074).

Immunofluorescence:
Primary antibodies: 1:10000 mouse anti-alpha-tubulin (Sigma T6199), 1:1000 rabbit anti-histone H3pS28 (Abcam, ab5169), 1:1000 mouse anti-emerin (Abcam ab204987), 1:1000 rabbit anti-Lamin B-receptor (Abcam, ab32535), 1:1000 rabbit anti-Lamin A (Abcam, ab26300), 1:1000 mouse anti-Lamin A/C (Santa Cruz sc-7292 (636)), 1:500 mouse anti-Elys (BioMatrix research, BMR00513), 1:1000 rabbit anti-Nup160 (Abcam, ab73293), 1:1000 mouse anti-Mab414 (Abcam, ab24609), 1:2000 rabbit anti-SON (Thermofisher, PA5-65107), 1:2000 mouse anti-NPM1 (Thermofisher, 60096-1), 1:1000 rabbit anti-Rad21 (Abcam, ab154769), 1:1000 rabbit anti-CTCF (Cell signaling, 2899), 1:1000 rabbit anti-RNAPolII pS2 (Abcam, ab5095). Secondary antibodies: 1:1000 goat anti-mouse IgG H+L Alexa Fluor 488 (Abcam, ab150113), 1:1000 goat anti-mouse IgG H+L Alexa Fluor 568 (Abcam ab175473), 1:1000 goat anti-rabbit IgG H&L Alexa Fluor 488 (Abcam ab15007), 1:1000 goat anti-rabbit IgG H&L Alexa Fluor 568 (Abcam, ab175471).

Validation

all previously published commercial antibodies

Eukaryotic cell lines

Policy information about cell lines and Sex and Gender in Research

Cell line source(s)	Engineered DLD-1 cell lines<br>Previously published and described in Supplementary methods.<br><br>The CRISPR/Cas9 system was used to endogenously target the RanGAP1, RCC1 (Aksenova et al. 2022, 2020), NUP93 (Regmi et al. 2020) and AAVS1 (Chu et al. 2015) genes. With the exception of nuclear import assays, all experiments described here employed human colorectal adenocarcinoma DLD-1 cells (ATCC CCL-221) expressing either RanGAP1 or Nup93 homozygously tagged with NeonGreen and an Auxin-Inducible Degron, Infra-Red protein (IFP)-tagged RCC1 and Tir1, as described previously. We refer to these cell lines as RanGAP1-AID and Nup93-AID.<br><br>For cell lines used in nuclear import assays, the sequences of MBP and mScarlet, were amplified by PCR from pMAL (NEB) and pmScarlet_alphaTubulin_C1 (Addgene, #85045), respectively. The NLS sequence was synthesized, and all fragments were inserted by Gibson reaction (E2611S, NEB) into the MCS of AAVS1_Puro_PGK1 vector (Addgene, #68375) through replacement of 3xFlagTwinStep-Tag. MBP-mScarlet-NLS was inserted into the AAVS1 locus in RanGAP1-AID and NUP93-AID DLD-1 cell lines.
Authentication	DNA from DLD-1 and CRISPR/Cas9-targeted cells was extracted with the Wizard® Genomic DNA Purification Kit (Promega). Clones were genotyped by PCR for homozygous insertion of tags with two sets of primers as described in Aksenova et al 2020 (RanGAP1) and Regmi et al 2020 (Nup93).
Mycoplasma contamination	Cell lines were routinely tested for mycoplasma infection and tested negative (MycoAlertTM Mycoplasma Detection Kit, Lonza).
Commonly misidentified lines<br>(See ICLAC register)	No commonly misidentified cell lines were used in this study.

Plants

Seed stocks	*Report on the source of all seed stocks or other plant material used. If applicable, state the seed stock centre and catalogue number. If plant specimens were collected from the field, describe the collection location, date and sampling procedures.*
Novel plant genotypes	*Describe the methods by which all novel plant genotypes were produced. This includes those generated by transgenic approaches, gene editing, chemical/radiation-based mutagenesis and hybridization. For transgenic lines, describe the transformation method, the number of independent lines analyzed and the generation upon which experiments were performed. For gene-edited lines, describe the editor used, the endogenous sequence targeted for editing, the targeting guide RNA sequence (if applicable) and how the editor was applied.*
Authentication	*Describe any authentication procedures for each seed stock used or novel genotype generated. Describe any experiments used to assess the effect of a mutation and, where applicable, how potential secondary effects (e.g. second site T-DNA insertions, mosiacism, off-target gene editing) were examined.*

Flow Cytometry

Plots

Confirm that:

☒ The axis labels state the marker and fluorochrome used (e.g. CD4-FITC).

☒ The axis scales are clearly visible. Include numbers along axes only for bottom left plot of group (a 'group' is an analysis of identical markers).

☒ All plots are contour plots with outliers or pseudocolor plots.

☒ A numerical value for number of cells or percentage (with statistics) is provided.

Methodology

Sample preparation	See supplementary methods. EtOH or FA/DSG fixation used for downstream application:<br><br>For cell cycle dynamics determinations:<br>Cells were collected at various points of mitotic exit. Adherent cells were dissociated with accutase (ThermoFisher Scientific, A11105-01) and pooled with non-adherent collected cells in order to assess the entire population. To assess the cell-cycle profile (DNA content), cell pellets were resuspended in 200ul PBS and fixed with 800µl of cold 100% ethanol. Cells were stored at −20°C for at least 24h. Approximately 1 million fixed cells were stained with 50 ug/ml propidium iodide (PI) (Thermo, P1304MP), diluted in 1 ml PBS containing 50 ug/ml RNaseA (Roche, 10109169001) and 0.1% Saponin, for 1 hour at room temperature. After staining, cells were spun, and pellets were resuspended in 1ml of PBS and passed through a 35 um filter (Falcon 352235).<br><br>For Hi-C sorting:<br>Cells were collected at various points of mitotic exit and fixed for Hi-C 3.0 (Lafontaine et al. 2021) with a few modifications to

facilitate cell sorting. Adherent cells collected 5 and 10 hours after prometaphase release, were dissociated with accutase. Prometaphase (t=0) and early (t=1.25-1.5h) released cells were directly collected by shake-off. Cell suspensions were pelleted and treated with accutase for an additional 5 minutes at room temperature to prevent aggregation and washed with HBSS (Thermofisher, 14025134). Fixation proceeded first with 1% formaldehyde (Fisher, BP531-25) in HBSS for 10 minutes, which was quenched with 0.125M Glycine for 5 minutes at room temperature and 15 minutes on ice. Next, cells were fixed with 3mM disuccinimidyl glutarate (DSG) in PBS for 40 minutes rotating at room temperature, followed by a second quenching with 0.125M Glycine. Fixed cells were washed twice with PBS + 0.1% BSA and snap-frozen in liquid nitrogen prior to staining for Fluorescence-activated cell sorting (FACS). In order to sort cells by DNA content, approximately 10 million fixed cells were stained with 50 ug/ml PI, diluted in 5 ml PBS containing 50 ug/ml RNaseA and 0.1% Saponin, for 1 hour at room temperature. Cells were then spun and washed with PBS prior to resuspension in 2 ml PBS + 0.1% BSA and passage through a 35 um filter.

Instrument

See supplementary methods. EtOH or FA/DSG fixation used for downstream application:

For cell cycle dynamics determinations:
Cells were collected at various points of mitotic exit. Adherent cells were dissociated with accutase (ThermoFisher Scientific, A11105-01) and pooled with non-adherent collected cells in order to assess the entire population. To assess the cell-cycle profile (DNA content), cell pellets were resuspended in 200ul PBS and fixed with 800μl of cold 100% ethanol. Cells were stored at −20°C for at least 24h. Approximately 1 million fixed cells were stained with 50 ug/ml propidium iodide (PI) (Thermo, P1304MP), diluted in 1 ml PBS containing 50 ug/ml RNaseA (Roche, 10109169001) and 0.1% Saponin, for 1 hour at room temperature. After staining, cells were spun, and pellets were resuspended in 1ml of PBS and passed through a 35 um filter (Falcon 352235).

For Hi-C sorting:
Cells were collected at various points of mitotic exit and fixed for Hi-C 3.0 (Lafontaine et al. 2021) with a few modifications to facilitate cell sorting. Adherent cells collected 5 and 10 hours after prometaphase release, were dissociated with accutase. Prometaphase (t=0) and early (t=1.25-1.5h) released cells were directly collected by shake-off. Cell suspensions were pelleted and treated with accutase for an additional 5 minutes at room temperature to prevent aggregation and washed with HBSS (Thermofisher, 14025134). Fixation proceeded first with 1% formaldehyde (Fisher, BP531-25) in HBSS for 10 minutes, which was quenched with 0.125M Glycine for 5 minutes at room temperature and 15 minutes on ice. Next, cells were fixed with 3mM disuccinimidyl glutarate (DSG) in PBS for 40 minutes rotating at room temperature, followed by a second quenching with 0.125M Glycine. Fixed cells were washed twice with PBS + 0.1% BSA and snap-frozen in liquid nitrogen prior to staining for Fluorescence-activated cell sorting (FACS). In order to sort cells by DNA content, approximately 10 million fixed cells were stained with 50 ug/ml PI, diluted in 5 ml PBS containing 50 ug/ml RNaseA and 0.1% Saponin, for 1 hour at room temperature. Cells were then spun and washed with PBS prior to resuspension in 2 ml PBS + 0.1% BSA and passage through a 35 um filter.

Software

FlowJo v10

Cell population abundance

10,000 total events typically recorded. Downsampling for plotting live events as indicated

Gating strategy

Live cell gating based on FSC-A/SSC-A distribution (see Supplementary Fig. 1).

For cell cycle determination:
Flow cytometry for the mitotic release timecourse was performed on a MACSQUANT set-up for at least three biological replicates. Analysis was performed using FlowJo software (v10) and plots reflect populations from a representative experiment gated for debris but not doublets and sampled to equal live cell event numbers.

For Hi-C sorting:
Propidium iodide-stained cell suspensions were sorted on a BD FACS Melody using the 561 nm laser for FSC, SSC, and PI. All populations were gated based on FSC/SSC to eliminate cell debris and cells sorted for either prometaphase (4n) or G1 (2n) DNA content were also subject to doublet discrimination. To enrich for telophase or cytokinesis, cells fixed 1.25 and 1.5 hours after mitotic release, respectively, were sorted based on doubled PI signal (DNA content) area and width. All sorted cells were collected in PBS containing 1% BSA and washed twice in PBS prior to snap-freezing.

☒ Tick this box to confirm that a figure exemplifying the gating strategy is provided in the Supplementary Information.
