# Supplementary material for: Interphase chromosome conformation is specified by distinct folding programmes inherited through mitotic chromosomes or the cytoplasm
Source: Nat Cell Biol. 2025 Dec 22;28(1):82–97. doi: 10.1038/s41556-025-01828-1 (PMC12807859; doi:10.1038/s41556-025-01828-1)
Supplement: Supplementary file 6 — Unprocessed western blots. [file 41556_2025_1828_MOESM6_ESM.pdf]

Figure 1b: Depletion of RanGAP1 or Nup93 during 5h mitotic exit (shown)

Replicate 1 quant.

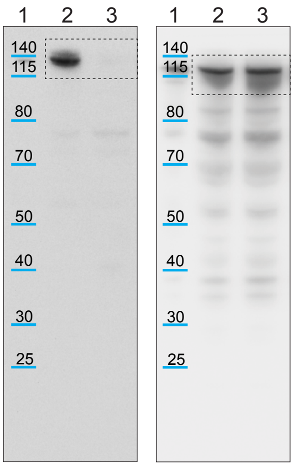

anti-RanGAP1 anti-vinculin

1 apparent MW
2 DLD1-RanGAP1-AID control
3 DLD1-RanGAP1-AID + IAA

Replicate 1 quant.

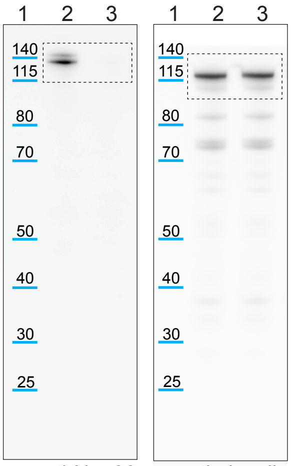

anti-Nup93 anti-vinculin

1 apparent MW
2 DLD1-Nup93-AID control
3 DLD1-Nup93-AID + IAA

Figure 1b: Additional blots used for quantification of RanGAP1 or Nup93 depletion during 5h mitotic exit

Replicate 2 quant.

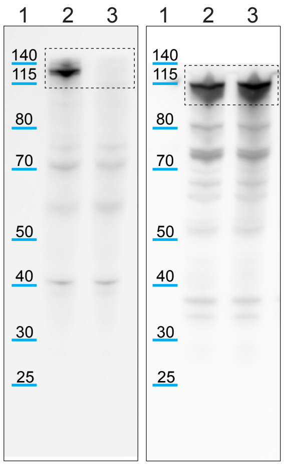

anti-RanGAP1 anti-vinculin

Replicate 3 quant.

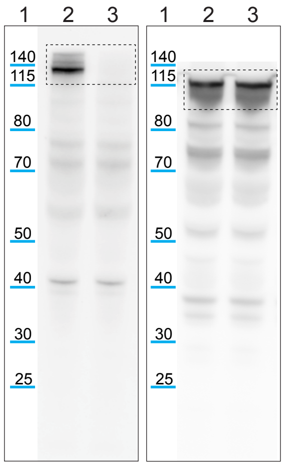

anti-RanGAP1 anti-vinculin

1 apparent MW
2 DLD1-RanGAP1-AID control
3 DLD1-RanGAP1-AID + IAA

Replicate 2 quant.

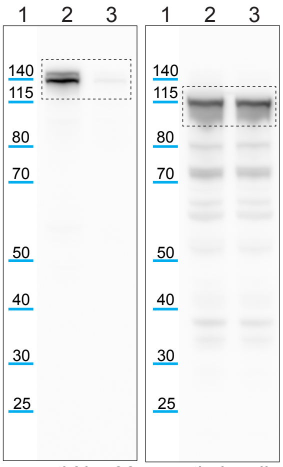

anti-Nup93 anti-vinculin

Replicate 3 quant.

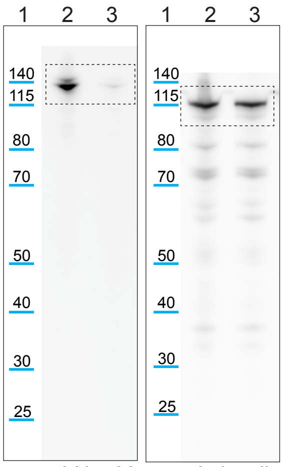

anti-Nup93 anti-vinculin

1 apparent MW
2 DLD1-Nup93-AID control
3 DLD1-Nup93-AID + IAA
