# Supplementary figures and images for: Interphase chromosome conformation is specified by distinct folding programmes inherited through mitotic chromosomes or the cytoplasm
Source: Nat Cell Biol. 2025 Dec 22;28(1):82–97. doi: 10.1038/s41556-025-01828-1 (PMC12807859; doi:10.1038/s41556-025-01828-1)

**Figure 5e: Use of BET protein inhibitors during mitotic exit in the presence of absence of RanGAP1**

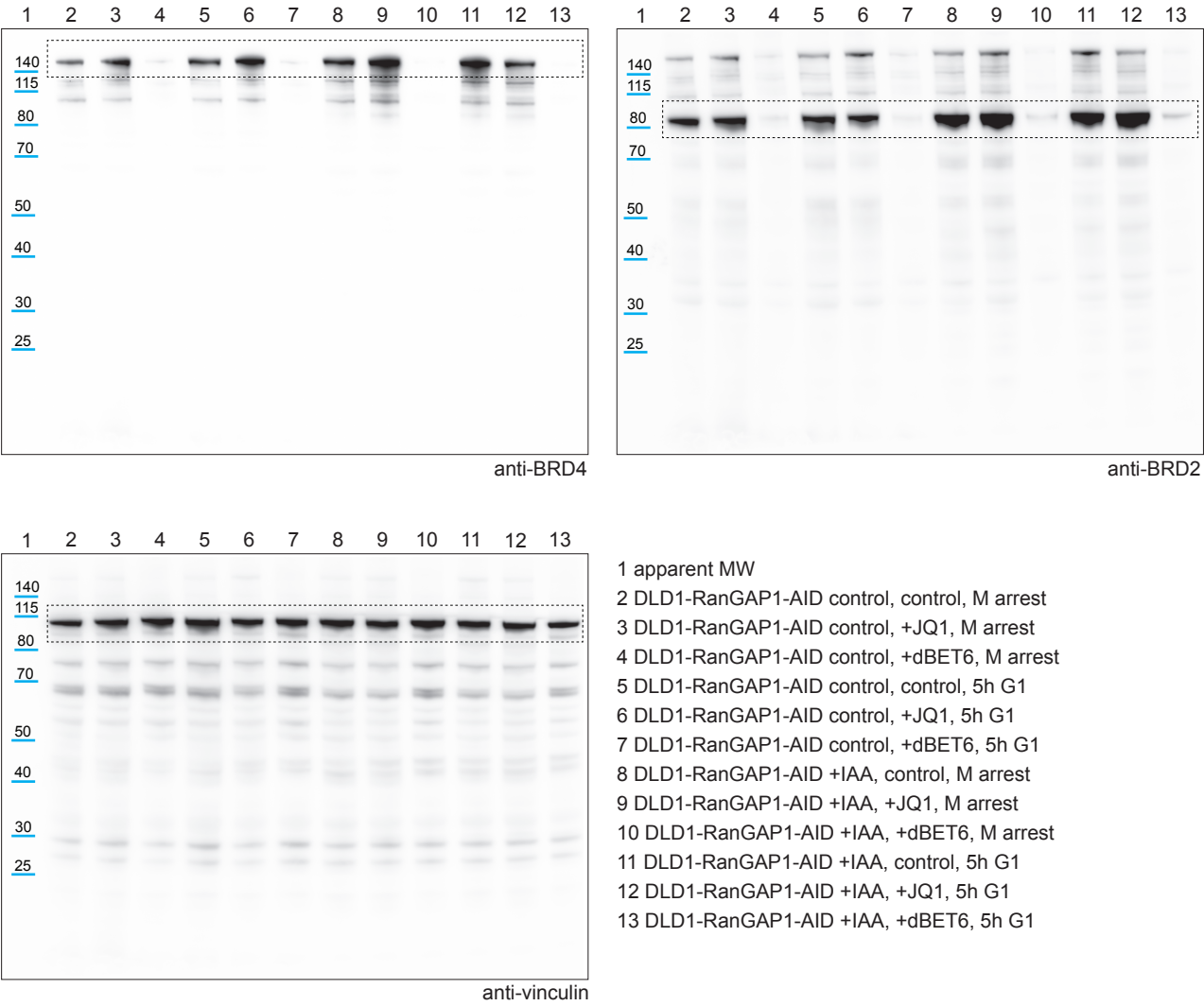

Supplement: Supplementary file 11 — Unprocessed western blots. [file 41556_2025_1828_MOESM11_ESM.pdf]

Supplementary Figure 1: wt and AID-tagged RanGAP1 and Nup93 cell lines

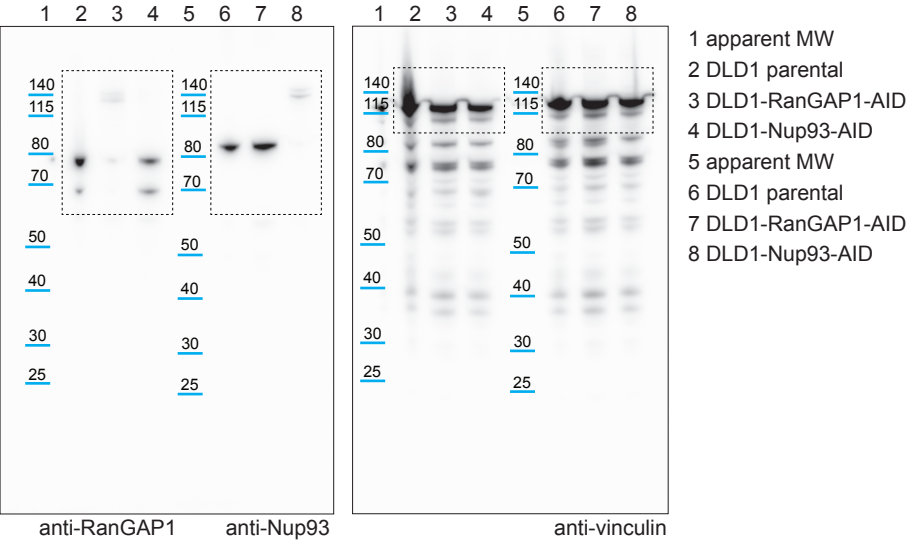

Supplement: Supplementary file 14 — Unprocessed western blots. [file 41556_2025_1828_MOESM14_ESM.pdf]
