# Supplementary material for: Interphase chromosome conformation is specified by distinct folding programmes inherited through mitotic chromosomes or the cytoplasm
Source: Nat Cell Biol. 2025 Dec 22;28(1):82–97. doi: 10.1038/s41556-025-01828-1 (PMC12807859; doi:10.1038/s41556-025-01828-1)
Supplement: Supplementary file 19 — Unprocessed western blots. [file 41556_2025_1828_MOESM19_ESM.pdf]

Extended Figure 9b: RanGAP1 depetion during G1

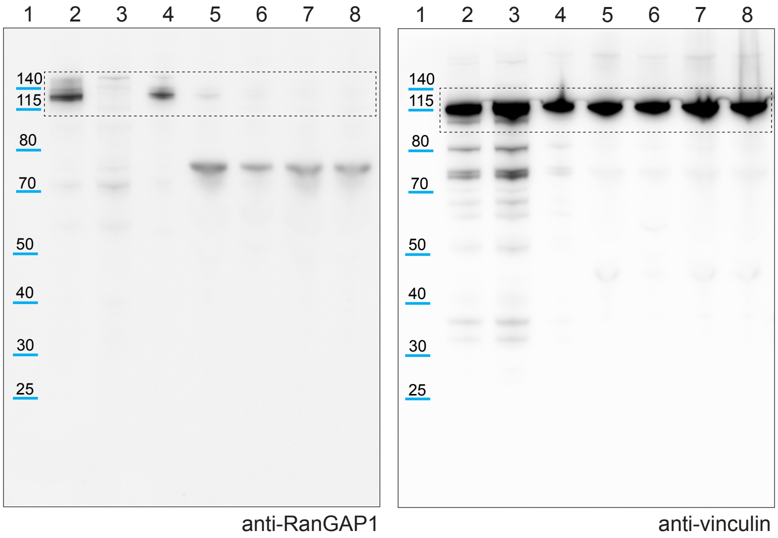

1 apparent MW
2 DLD1-RanGAP1-AID control, t=0 (mitotic arrest)
3 DLD1-RanGAP1-AID +IAA, t=0 (mitotic arrest)
4 DLD1-RanGAP1-AID control, t=3.5h release
5 DLD1-RanGAP1-AID +IAA @ 3.5h, t=4.0h release
6 DLD1-RanGAP1-AID +IAA @ 3.5h, t=4.5h release
7 DLD1-RanGAP1-AID +IAA @ 3.5h, t=5.0h release
8 DLD1-RanGAP1-AID +IAA @ 3.5h, t=5.5h release
